# Supplementary material for: Incidence, Severity and Clinical Factors Associated with Hypotension in Patients Admitted to an Intensive Care Unit: A Prospective Observational Study
Source: J Clin Med. 2022 Nov 18;11(22):6832. doi: 10.3390/jcm11226832 (PMC9696980; doi:10.3390/jcm11226832)
Supplement: Supplementary file 1 [file jcm-11-06832-s001.zip › jcm-1951853-supplementary.pdf]

Table of contents

Supplements 2
Supplemental Figure S1. Flowchart of patient inclusion 2
Supplemental Table S1. Signal quality index criteria based on arterial waveform features within a beat (top part) and variations between beats (bottom part) 3
Supplemental Table S2. All baseline characteristics 4
Supplemental Table S3. Subgroups of hypotension based on absolute mean arterial pressure threshold 8
Supplemental Table S4. Univariable binary logistic regression analysis (mean arterial pressure < 65 mmHg) 11
Supplemental Table S5. Univariable binary logistic regression analysis (time-weighted average) 12
Supplemental Table S6. Incidence and severity of hypotension in all intensive care patients 14

Supplements

Supplemental Figure S1. Flowchart of patient inclusion

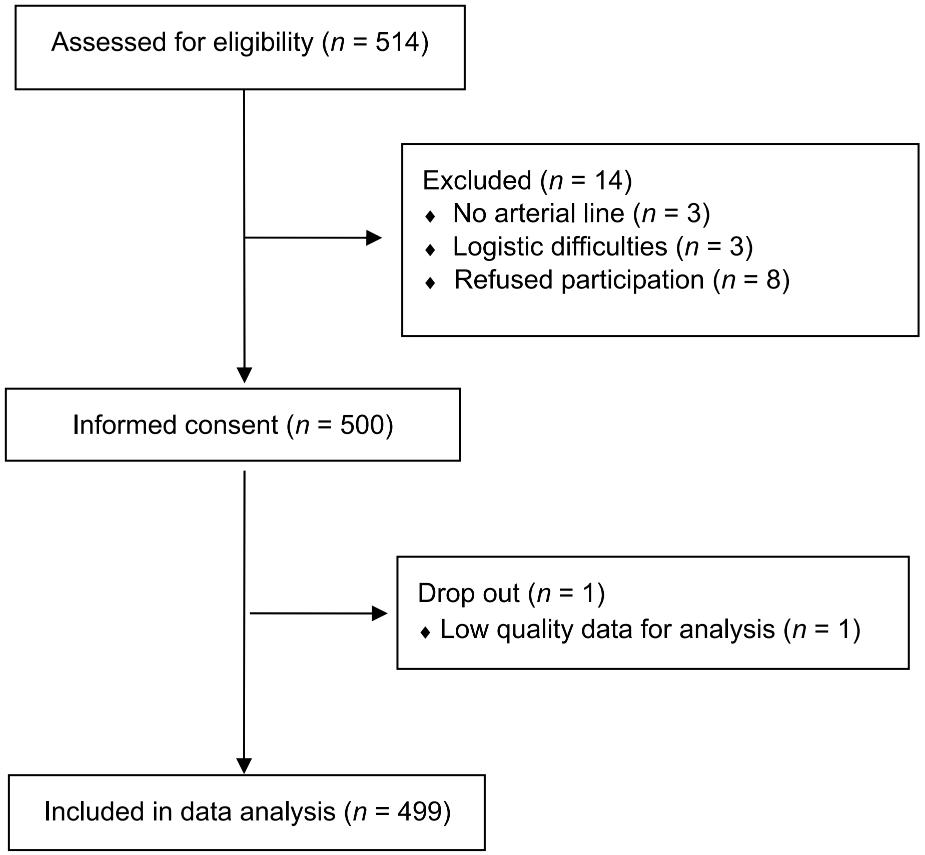

Supplemental Table S1. Signal quality index criteria based on arterial waveform features within a beat (top part) and variations between beats (bottom part)

Feature Abnormality criteria	Abnormality criteria
Systolic blood pressure (SBP)	SBP > 300 mmHg
Diastolic blood pressure (DBP)	DBP < 20 mmHg
Mean arterial pressure (MAP)	MAP < 30 or MAP > 200 mmHg
Heart rate (HR)	HR < 20 or HR > 200 bpm
Pulse pressure (PP)	PP < 20 mmHg
Noise level (w)	w < 500 mmHg/sec
Difference in systolic pressure (SBP_k – SBP_k-1)	Delta SBP > 20 mmHg
Difference in diastolic pressure (DBP_k – DBP_k-1)	Delta DBP > 20 mmHg
Difference in beat duration (Tk – Tk-1)	Delta T > 2/3 sec

The onset of a single heartbeat was detected by an algorithm based on the open-source beat detection algorithm that Zong and colleagues of Harvard MIT made in 2003. When the beat was detected, it was determined whether this beat has a valid arterial pressure signal. Since further signal analysis will probably not be accurate if the beat contains noise and artefacts, beats with bad signal quality were removed. The signal quality determination is mostly based on the robust method of Sun et al. and Asgari et al. In 2006 Sun and colleagues introduced the signal abnormality index, based on the presence of noise and physiological and beat-to-beat variation criteria. The signal quality index (SQI), an algorithm tested on 246 arterial blood pressure segments and compared to the findings of a human expert. The index reached a sensitivity of 1.00, specificity of 0.91, positive predictive value of 0.73 and a negative predictive value of 1.00.In this table, all abnormality criteria can be found, the top part consists of all physiological criteria and the bottom part of the beat-to-beat variation criteria. The boundaries were based upon the ones used by Sun and adapted for this particular data when necessary to eliminate all possible artefacts, noise and bad beats. The pressure parameters were calculated using max, min and mean functions on the arterial blood pressure signal. Heart rate is 60 divided by the heart cycle duration and PP is the difference between SBP and DBP. To identify high frequency noise, the noise level w was expressed as the sum of the negative slopes in the signal. Since systolic contractility leads to strong positive slopes, only the negative slopes are used for detection of noise. If any of the abnormality criteria is met, the SQI is set to zero, otherwise the SQI is one. Like Asgari et al. did in their research, the SQI is modified with the condition that the beat-to-beat variation could only be taken into account when the previous beat was labelled as valid. When the previous beat was invalid only the top six abnormality criteria were applied. With this modification the true positive rate increased from 93.63% to 95.57% in their research. Only when the SQI was high, the base value for adaptive thresholding in the beat detection algorithm was updated.

Supplemental Table S2. All baseline characteristics

**Baseline parameters**	**All patients** *n* = 499
Sex, male, *n* (%)	327 (6)
Age, years, mean (sd)	61 (14)
Number of patients older than 65 years, *n* (%)	221 (44)
Weight (kg), mean (sd)	82.97 (19.5)
Height (cm), mean (sd)	174 (9.9)
BMI, mean (sd)	27 (6)
SOFA score, mean (sd)	10 (3)
Not calculated, *n* (%)	79 (15.8)
**Measurement details**	
Blood pressure monitoring time per patient (minutes), median [IQR]	441 [411 – 962]
Signal quality percentage per patient, median [IQR]	98.0 [94.6 – 99.0]
Number of daytime measurements, *n* (%)	305 (61)
Number of night-time measurements, *n* (%)	194 (39)
Number of patients with a MAP<65mmHg, *n* (%)	376 (75)
**Medical history**	
Myocardial infarction, *n* (%)	70 (14)
Atrial fibrillation, *n* (%)	57 (11)
Decompensated heart failure, *n* (%)	20 (4)
Hypertension, *n* (%)	146 (29)
History of cardiac surgery, *n* (%)	14 (3)
Cardiovascular disease (other), *n* (%)	94 (19)
COPD, *n* (%)	26 (5)
Asthma, *n* (%)	17 (3)
Obese, *n* (%)	18 (4)
OSAS, *n* (%)	19 (4)
Diabetes mellitus type 1, *n* (%)	4 (1)
Diabetes mellitus type 2, *n* (%)	81 (16)
Hypothyroidism, *n* (%)	11 (2)
TIA, *n* (%)	24 (5)

CVA, *n* (%)	24 (5)
History of neurosurgery, *n* (%)	6 (1)
Gastrointestinal disease, *n* (%)	76 (15)
Renal disease and insufficiencies, *n* (%)	35 (7)
Oncological disease, *n* (%)	23 (5)
Alcohol or drug abuse, *n* (%)	23 (5)
**Cardiovascular home medication**	
ACE inhibitors, *n* (%)	97 (19)
Beta-blockers, *n* (%)	154 (31)
Angiotensin II inhibitors, *n* (%)	30 (6)
Calcium channel blockers, *n* (%)	40 (8)
Diuretics, *n* (%)	107 (21)
Statins, *n* (%)	191 (38)
**Metabolic disease home medication**	
Thyrax, *n* (%)	17 (3)
Corticosteroids, *n* (%)	29 (6)
Oral antidiabetics, *n* (%)	69 (14)
Insulin, *n* (%)	45 (9)
**Pulmonary home medication**	
Beta 2 agonists; bronchodilators, *n* (%)	14 (3)
Inhalation corticosteroids, *n* (%)	150 (30)
**Other home medication**	
Platelet aggregation inhibitors, *n* (%)	25 (5)
Vitamin K inhibitors, *n* (%)	7 (1)
Anti-epileptic drugs, *n* (%)	166 (33)
Proton pump inhibitors, *n* (%)	34 (7)
Paracetamol, *n* (%)	18 (4)
NSAIDs, *n* (%)	20 (4)
Opioids, *n* (%)	45 (9)
Benzodiazepines, *n* (%)	275 (55)
**Reason of ICU admission**	
Other, *n* (%)	26 (5)
Sepsis, *n* (%)	38 (8)
Post cardiac arrest, *n* (%)	40 (8)

Post cardiac surgery, *n* (%)	199 (40)
Cardiac shock/other cardiac, *n* (%)	19 (4)
Pneumonia-ARDS, *n* (%)	19 (4)
Respiratory other, *n* (%)	38 (8)
Intracranial bleeding (SAB), *n* (%)	51 (10)
Neurogenic shock, *n* (%)	3 (1)
Neurological other, *n* (%)	28 (6)
Trauma, *n* (%)	15 (3)
Postoperative non cardiac surgery, *n* (%)	17 (3)
**Assigned shock groups**	
Cardiogenic shock, *n* (%)	66 (13)
Distributive shock, *n* (%)	94 (19)
Hypovolemic shock, *n* (%)	12 (2)
Obstructive shock, *n* (%)	2 (0.4)
Combination type of shock, *n* (%)	32 (6)
Non-shock classification, *n* (%)	293 (59)
**Vitals**	
Temperature °C , median [Q1-Q3]	36.8 [36.2-37.4]
Lactate (mmol/L), median [Q1-Q3]	1.7 [1.3-2.4]
Diuresis (ml/kg/h), median [Q1-Q3]	0.8 [0.6-1.4]
Hemoglobine (mmol/L),), median [Q1-Q3]	6.6 [5.7-7.4]
Saturation (%), median [Q1-Q3]	93 [74-95]
**Vasoactive medication during measurements**	
Norepinephrine, *n* (%)	271 (54)
Milrinone, *n* (%)	17 (3)
Dobutamine, *n* (%)	14 (3)

**Mechanical ventilation, *n* (%)**	358 (72)
**Type of mechanical ventilation**	
ASV, *n* (%)	181 (36)
PCV, *n* (%)	99 (20)
VCV, *n* (%)	2 (0.4)
PSV, *n* (%)	59 (12)
NIV, *n* (%)	6 (1)
CPAP, *n* (%)	6 (1)
Oxygen support *n* (%)	51 (10)
Spontaneous, *n* (%)	43 (9)

Statistic presented as mean (standard deviation), median [first quartile, third quartile] or number of patients (%).
Abbreviations: MAP, mean arterial pressure; COPD, chronic obstructive pulmonary disease; CABG, coronary artery bypass graft; BMI, body mass index; TIA, transient ischemic attack, CVA, cerebral vascular accident; ACE, angiotensine converterend enzyme; SOFA, sequential organ failure assessment; OSAS, obstructive sleep apnoea syndrome; ASV, assisted support ventilation, PCV= pressure controlled ventilation; VCV,volume controlled ventilation; PSV, pressure support ventilation; NIV, non-invasive ventilation; CPAP,continuous positive airway pressure

Supplemental Table S3. Subgroups of hypotension based on absolute mean arterial pressure threshold

**Baseline parameters**	**MAP ≥ 65 mmHg** *n*<br>*n* = 123 (25%)	**MAP < 65-60 mmHg**<br>*n* = 67 (13%)	**MAP < 60-55 mmHg**<br>*n* = 121 (24%)	**MAP < 55-50 mmHg**<br>*n* = 89 (18%)	**MAP < 50-45 mmHg**<br>*n* = 53 (11%)	**MAP < 45 mmHg**<br>*n* = 46 (9%)	***p*-value**
Sex, male, *n* (%)	71 (58)	44 (66)	77 (64)	60 (67)	41 (77)	34 (74)	0.172
Age, years, mean (sd)	57 (14)[‡]	63 (14)	63 (13)	59 (14)[†]	61 (17)	68 (12)[‡†]	**<0.001**
BMI, mean (sd)	28 (6)	28 (7)	27 (7)	27 (6)	26 (4)	28 (5)	0.086
SOFA score, median [Q1-Q3]	10 [7,12]	10 [8,12]	10 [8,12]	9 [7,12]	10 [7,12]	11 [8,11]	0.537
Day-time measurements, *n* (%)	87 (71)	38 (57)	70 (58)	56 (63)	28 (53)	26 (57)	0.111
**Medical history**							
Myocardial infarction, *n* (%)	11 (9)	11 (16)	17 (14)	12 (14)	12 (23)	7 (15)	0.736
Hypertension, *n* (%)	37 (30)	22 (33)	37 (31)	24 (27)	16 (30)	10 (22)	0.841
Pulmonary disease, *n* (%)	4 (3)	5 (8)	7 (6)	3 (3)	3 (6)	4 (9)	0.750
Diabetes mellitus type II, *n* (%)	15 (12)	16 (24)	21 (17)	12 (14)	6 (11)	11 (24)	0.162
CVA, *n* (%)	6 (5)	1 (2)	9 (7)	4 (5)	1 (2)	3 (7)	0.449
Gastrointestinal disease, *n* (%)	19 (16)	9 (13)	28 (23)	10 (11)	5 (9)	5 (11)	0.121
Renal insufficiencies, *n* (%)	6 (5)	7 (10)	13 (11)	3 (3)	5 (9)	1 (2)	0.292
Oncological disease, *n* (%)	7 (6)	4 (6)	7 (6)	2 (2)	3 (6)	-	0.117
Alcohol, smoking or drug abuse, *n* (%)	9 (7)	2 (3)	1 (1)	5 (6)	2 (4)	4 (9)	0.872
**Reason of ICU admission**							
OHCA, *n* (%)	4 (3)	12 (18)	5 (4)	5 (6)	4 (8)	1 (2)	**<0.001**
IHCA, *n* (%)	1 (1)	3 (5)	2 (2)	3 (3)	-	-	0.267
Post cardiac surgery, *n* (%)	32 (26)	27 (40)	54 (45)	36 (40)	26 (49)	23 (50)	**0.013**
Cardiac shock/other cardiac, *n* (%)	2 (2)	1 (2)	6 (5)	2 (2)	5 (9)	3 (7)	0.107
Pneumonia, *n* (%)	5 (4)	4 (6)	5 (4)	4 (5)	1 (2)	-	0.614
Respiratory other, *n* (%)	10 (8)	3 (5)	8 (7)	8 (9)	5 (9)	4 (9)	0.885
Intracranial bleeding (SAB), *n* (%)	4 (3)	4 (6)	10 (8)	6 (7)	3 (6)	1 (2)	0.194
Neurological other, *n* (%)	10 (8)	6 (9)	4 (3)	5 (6)	-	4 (9)	**<0.001**
Sepsis, *n* (%)	12 (10)	2 (3)	14 (12)	8 (9)	2 (4)	-	0.057
**Assigned shock groups**							
Cardiogenic shock, *n* (%)	4 (3)	11 (16)	14 (12)	17 (19)	10 (19)	10 (22)	**0.002**
Distributive shock, *n* (%)	20 (16)	8 (12)	30 (25)	18 (20.	8 (15)	9 (20)	0.305
Hypovolemic shock, *n* (%)	1 (1)	2 (3)	4 (3)	3 (3)	1 (2)	1 (2)	0.815
Obstructive shock, *n* (%)	1 (1)	-	-	-	1 (2)	-	0.446

Combination type of shock, *n* (%)	8 (7)	5 (8)	5 (8)	5 (6)	4 (8)	6 (13)	0.343
Non-shock classification, *n* (%)	88 (72)	41 (61)	69 (57)	46 (52)	29 (55)	20 (445)	**0.007**
**Clinical data**							
Lactate (mmol/L), median [Q1-Q3]	1.5 [1.2,1.9]	1.4 [0.9,1.9]	1.4 [1.6,2.0]	1.4 [1.1,2.1]	1.3 [1.0,2.4]	1.7 [1.0,2.3]	0.397
Missing, n (%)	57 (46.3)	19 (28.4)	36 (29.8)	19 (21.3)	8 (15.1)	11 (23.9)	
Diuresis (ml/kg/h), median [Q1-Q3]	0.9 [0.6,1.5]	0.9 [0.6,1.6]	0.9 [0.6,1.2]	0.9 [0.5,1.6]	0.9 [0.6,1.3]	0.8 [0.6,1.2]	0.916
Missing, n (%)	5 (4.1)	-	6 (5.0)	4 (4.5)	-	1 (2.2)	
Haemoglobin (mmol/L),median [Q1-Q3]	6.9 [5.9,7.8]	6.8 [5.7,7.8]	6.4 [5.6,7.3]	6.7 [5.8,7.4]	6.5 [5.6,7.3]	6.3 [5.5,7.4]	0.165
Missing, n (%)	21 (17.1)	3 (4.5)	16 (13.2)	6 (6.7)	1 (1.9)	6 (13.0)	
Saturation (%), median[Q1-Q3]	94 [91,96]	92 [72,95]	93 [72,95]	92 [68,95]	93 [73,95]	93 [68,95]	0.080
Missing, n (%)	22 (17.9)	3 (4.5)	16 (13.2)	6 (6.7)	1 (1.9)	6 (13.0)	
Maximum Norepinephrine dose (mcg/kg/min), mean (sd)	0.17 (0.15)	0.12 (0.11)[∞]	0.18 (0.19)	0.23 (0.19)[∞]	0.18 (0.17)	0.20 (0.17)	**0.027**
Minimum Norepinephrine dose (mcg/kg/min), mean (sd)	0.11 (0.15)	0.04 (0.05)	0.08 (0.16)	0.09 (0.12)	0.08 (0.14)	0.08 (0.11)	0.728
No treatment with Norepinephrine, *n* (%)	61 (50)	26 (39)	50 (41)	33 (37)	15 (28)	10 (22)	0.102
Mechanical ventilation, *n* (%)	83 (68)	48 (72)	83 (69)	68 (76)	39 (74)	35 (76)	0.736
**Haemodynamic data**							
Number of events per patients, median [Q1-Q3]	- [# ¥®‡]	2.00 [1.00-4.00][Ø]	2.00 [1.00-5.00][#]	1.00 [1.00-2.50][¥]	1.00 [1.00-2.00][®Ø]	1.00 [1.00-2.00][‡]	**<0.001**
Total duration of events per patient (min), median [Q1-Q3]	- [¥®]	4.99 [1.59-20.05][•Ø]	5.35 [1.52-25.37][•]	1.65 [0.48-4.93][¥]	1.19 [0.42-2.95][®Ø]	1.15 [0.39-3.51]	**<0.001**
Total duration of events per patient (%), median [Q1-Q3]	- [×#¥®‡]	1.01 [0.26-4.42][×]	1.20 [0.25-3.26][#]	0.25 [0.08-0.82][¥]	0.14 [0.07-0.37][®]	0.25 [0.05-0.49][‡]	**<0.001**
TWA per patient (mmHg), median [Q1-Q3]	- [×#¥®‡]	0.07 (0.11)[×]	0.07 (0.11)[#]	0.03 (0.06)[¥]	0.01 (0.02)[®]	0.09 (0.34)[‡]	**<0.001**
Cardiac output (L/min), median [Q1-Q3]	6.1 [4.9-7.6]	5.8 [4.6-7.5]	5.6 [4.7-6.9]	5.4 [4.4-6.6]	5.9 [4.7-6.8]	5.2 [4.4-6.3]	0.378
Cardiac index (L/min/m2), median [Q1-Q3]	3.0 [2.4-3.8]	3.1 [2.4-3.8]	2.8 [2.4-3.3]	2.7 [2.3-3.4]	3.1 [2.6-3.5]	2.7 [2.3-3.1]	0.163
Stroke volume (ml), median [Q1-Q3]	76 [60-93]	72 [63-85]	70 [56-85]	67 [51-81]	78 [61-93]	66 [49-82]	0.128
Stroke volume index (ml/m2), median [Q1-Q3]	39 [31-46]	38 [31-46]	35 [30-41][¢]	34 [28-40]	37 [33-45][¢¤]	33 [27-41][¤]	**0.003**

Stroke volume variation (%), median [Q1-Q3]	12.4 [9.3-15.3]	13.4 [10.0-17.8]	14.1 [9.8-18.0]	12.8 [10.4-17.5]	12.5 [10.1-16.7]	14.5 [11.7-19.0]	0.453
Heart rate (beats/min), median [Q1-Q3]	85 [73- 96]	84 [72-95]	85 [74-96]	87 [72-101]	80 [69-95]	81 [69-91]	0.993
Pulse pressure variation (%), median [Q1-Q3]	10.0 [8.2-13.7]	13.0 [8.4-17.5]	11.9 [8.3-17.2]	12.7 [8.8-15.7]	11.0 [8.4-16.0]	13.6 [9.2-19.3]	0.201
Systolic BP (mmHg), median [Q1-Q3]	143 [125-168]	121 [107-134]	113 [106-124]	110 [100-121]	117 [106-129]	112 [102-124]	0.153
Diastolic BP (mmHg), median [Q1-Q3]	69 [64-75]×#¥®‡	59 [56-64]×	56 [53-60]#	53 [49-58]¥	53 [48-57]®	54 [49-58]‡	**<0.001**
Average MAP (mmHg), median [Q1-Q3]	93 [84-106]	78 [74-83]	73 [71-79]	71 [67-74]	70 [67-77]	71 [66-77]	0.531
Contractility (dPdt (mmHg/s), median [Q1-Q3]	1279 [965-1642]	1021 [776-1359]	892 [705-1120]	931 [720-1207]	1084 [718-1349]	948 [702-1386]	0.347

Abbreviations: BMI, body mass index; BP, blood pressure; CVA, cerebral vascular accident; IHCA, in hospital cardiac arrest; MAP, mean arterial pressure; OHCA, out of hospital cardiac arrest; SOFA, sequential organ failure assessment; TIA, transient ischemic attack; TWA, time weighted average.
Statistic presented as mean (standard deviation), median [first quartile, third quartile] or number of patients (%).
Group differences were tested with Kruskal Wallis, One-way ANOVA or Chi square test/Fisher exact. Post hoc testing (normal distribution post hoc testing with Games-Howell (not equal variances) or Tukey (equal variances) en if non-normal distribution with Mann-Whitney U). Significance values have been adjusted by the bonferroni correction for multiple tests.The significance level is 05.
Asymptotic post hoc significances (2-sided tests) are displayed with:
× significant difference MAP >65mmHg vs MAP 65-60mmHg
significant difference MAP >65mmHg vs MAP 60-55mmHg
¥ significant difference MAP >65mmHg vs MAP 55-50mmHg
® significant difference MAP >65mmHg vs MAP 50-45mmHg
‡ significant difference MAP >65mmHg vs MAP <45mmHg
● significant difference MAP 65-60mmHg vs MAP 60-55mmHg
∞ significant difference MAP 65-60mmHg vs MAP 55-50mmHg
Ø significant difference MAP 65-60mmHg vs MAP 50-45mmHg
¢ significant difference MAP 60-55mmHg vs MAP 50-45mmHg
† significant difference MAP 55-50mmHg vs MAP <45mmHg
¤ significant difference MAP 50-45mmHg vs MAP <45mmHg

Supplemental Table S4. Univariable binary logistic regression analysis (mean arterial pressure < 65 mmHg)

		95% CI		
**Covariate**	**Odds ratio**	**Lower**	**Upper**	***p*-value**
Age	1.023	1.009	1.037	**0.002**
Sex (male)	1.562	1.028	2.374	**0.037**
Height (cm)	2.230	0.287	17.332	0.443
Weight (kg)	0.992	0.982	1.002	0.113
BMI	0.968	0.936	1.001	**0.059**
Night measurement	1.842	1.184	2.866	**0.007**
History of myocardial infarction	1.622	0.803	3.278	0.178
History of diabetes mellitus type II	1.533	0.840	2.798	0.164
History of renal insufficiencies	1.378	0.549	3.458	0.495
Reason of ICU admission: post cardiac surgery	2.272	1.447	3.568	**<0.001**
Cardiogenic shock classification	5.874	2.091	16.501	**<0.001**
Distributive shock classification	1.262	0.734	2.170	0.400
Hypovolemic shock classification	3.677	0.470	28.77	0.215
Non-shock classification	0.453	0.291	0.706	**<0.001**
Saturation (%) during measurement	0.974	0.955	0.992	**0.006**
Maximum Norepinephrine dose (mcg/kg/min)	2.347	0.367	15.012	0.368
Minimum Norepinephrine dose (mcg/kg/min)	0.237	0.038	1.962	0.197
Treatment with Norepinephrine	1.763	1.169	2.658	**0.007**
Length of stay ICU (days)	0.991	0.973	1.009	0.310
Length of stay in hospital (days)	0.996	0.986	1.006	0.384
Died during ICU admission	1.588	0.819	3.082	0.171
Died during hospital admission	1.368	0.777	2.407	0.277

Supplemental Table S5. Univariable binary logistic regression analysis (time-weighted average)

		95% CI		
**Covariate**	**Odds ratio**	**Lower**	**Upper**	***p*-value**
Sex, male	1.202	0.809	1.785	0.363
Age, years	1.009	0.995	1.022	0.210
SOFA score	1.099	1.028	1.176	**0.006**
Temperature °C	0.832	0.661	1.048	0.118
History of cardiovascular disease (other)	1.227	0.769	1.957	0.391
History of hypertension	1.097	0.731	1.647	0.656
History of diabetes mellitus type 2	1.286	0.786	2.104	0.317
History of renal disease and insufficiencies	1.147	0.561	2.346	0.707
Reason of ICU admission: IHCA	7.219	1.483	35.144	**0.014**
Reason of ICU admission: post cardiac surgery	1.910	1.308	2.788	**<0.001**
Reason of ICU admission: pneumonia	0.603	0.757	0.265	2.161
Reason of ICU admission: Intracranial bleeding (SAB)	0.149	0.053	0.421	**<0.001**
Reason of ICU admission: other neurological disease	0.225	0.067	0.755	**0.016**
Reason of ICU admission: sepsis	1.493	0.762	2.926	0.243
Cardiogenic shock classification	2.766	1.635	4.682	**<0.001**
Distributive shock classification	1.809	1.144	2.860	**0.011**
Combination type of shock classification	2.395	1.165	4.925	**0.018**
Non-shock classification	0.314	0.214	0.462	**<0.001**
Lactate (mmol/L) during measurement	1.170	1.006	1.363	**0.042**
Diuresis (ml/kg/h) during measurement	0.697	0.543	0.895	**0.005**
Saturation (%) during measurement	0.976	0.963	0.963	**<0.001**
Haemoglobin (mmol/L) during measurement	0.803	0.689	0.936	**0.005**
Norepinephrine max dose (mcg/kg/min) during measurement	17.567	3.736	82.593	**<0.001**
Norepinephrine min dose (mcg/kg/min) during measurement	33.579	4.028	279.919	**<0.001**
Length of stay ICU (days)	0.986	0.965	1.007	0.195

Length of stay in hospital (days)	1.000	0.990	1.010	0.965
Died during ICU admission	1.638	0.966	2.777	0.067
Died during hospital admission	1.445	0.899	2.322	0.129

Abbreviations: SOFA, sequential organ failure assessment.

Severe TWA group vs Mild-Moderate TWA group

Supplemental Table S6. Incidence and severity of hypotension in all intensive care patients

**Quality of continuous blood pressure data for analysis of all patients**	
Total number of patients with correct data, *n* (%)	499 (100)
Monitoring time per patient (min), median [Q1-Q3]	441 [411-962]
Good signal quality (%), median [Q1-Q3]	98 [95-99]
Average MAP per patient (mmHg) , median [Q1-Q3]	76 [70-84]
Minimal MAP per patient (mmHg) , median [Q1-Q3]	47 [42-54]
**MAP ≤ 65 mmHg**	
Number of patients with hypotension (MAP < 65 mmHg) , *n* (%)	376 (75.4)
Number of events per patients,median [Q1-Q3]	5 [1-12]
Total duration of events per patient (min), median [Q1-Q3]	30.56 [0.17-143.52]
Total duration of events per patient (%), median [Q1-Q3]	5.6 [0.0-25.3]
TWA per patient (mmHg), median [Q1-Q3]	0.22 [0.0-0.86]
Total number of hypotensive events (MAP < 65 mmHg)	4090
Duration per event (min), median [Q1-Q3]	3.60 [0.90 -12.77]
AUT per event (mmHg*min), median [Q1-Q3]	9.73 [2.36-42.70]
Average MAP per event (mmHg), median [Q1-Q3]	61.28 [59.78-62.52]
Minimum MAP per event (mmHg), median [Q1-Q3]	54.46 [50.19-58.11]
**MAP ≤ 60 mmHg**	
Number of patients with MAP ≤ 60 mmHg, *n* (%)	309 (61.9)
Number of events per patients, median [Q1-Q3]	2 [0-6]
Total duration of events per patient (min), median [Q1-Q3]	3.25 [0-25.65]
Total duration of events per patient (%), median [Q1-Q3]	0.53 [0-4.15]
TWA per patiënt (mmHg), median [Q1-Q3]	0.02 [0-0.13]
Total number of events (MAP ≤ 60mmHg)	2200
Duration per event (min), median [Q1-Q3]	2.31 [0.62-6.65]
AUT per event (mmHg*min), median [Q1-Q3]	5.83 [1.54-20.22]
Average MAP per event (mmHg), median [Q1-Q3]	56.67 [55.12-57.81]
Minimum MAP per event (mmHg), median [Q1-Q3]	51.11 [47.26-54.10]
**MAP ≤ 55 mmHg**	
Number of patients with MAP ≤ 55 mmHg, *n* (%)	188 (37.7)
Number of events per patients, median [Q1-Q3]	0 [0-1]

Total duration of events per patient (min), median [Q1-Q3]	0 [0-2.03]
Total duration of events per patient (%), median [Q1-Q3]	0 [0-0.27]
TWA per patiënt (mmHg), median [Q1-Q3]	0 [0-0.01]
Total number of events (MAP ≤ 55 mmHg)	741
Duration per event (min), median [Q1-Q3]	1.45 [0.43-3.74]
AUT per event (mmHg*min), median [Q1-Q3]	3.93 [1.32-13.23]
Average MAP per event (mmHg), median [Q1-Q3]	51.67 [50.05-52.73]
Minimum MAP per event (mmHg), median [Q1-Q3]	46.71 [43.31-49.37]
**MAP ≤50mmHg**	
Number of patients with MAP ≤ 50 mmHg, *n* (%)	99 (19.8)
Number of events per patients, median [Q1-Q3]	0 [0-0]
Total duration of events per patient (min), median [Q1-Q3]	0 [0-0]
Total duration of events per patient (%), median [Q1-Q3]	0 [0-0]
TWA per patiënt (mmHg), median [Q1-Q3]	0 [0-0]
Total number of events (MAP ≤ 50 mmHg)	228
Duration per event (min), median [Q1-Q3]	1.18 [0.38-2.47]
AUT per event (mmHg*min), median [Q1-Q3]	3.43 [1.18-9.34]
Average MAP per event (mmHg), median [Q1-Q3]	46.35 [44.42-47.82]
Minimum MAP per event (mmHg) , median [Q1-Q3]	42.00 [38.01-45.21]
**MAP ≤ 45 mmHg**	
Number of patients with MAP ≤ 45 mmHg, *n* (%)	46 (9.2)
Number of events per patients, median [Q1-Q3]	0 [0-0]
Total duration of events per patient (min) , median [Q1-Q3]	0 [0-0]
Total duration of events per patient (%), median [Q1-Q3]	0 [0-0]
TWA per patient (mmHg), median [Q1-Q3]	0 [0-0]
Total number of events (MAP ≤45mmHg)	78
Duration per event (min), median [Q1-Q3]	0.87 [0.35-2.28]
AUT per event (mmHg*min), median [Q1-Q3]	2.97 [1.08-8.49]
Average MAP per event (mmHg), median [Q1-Q3]	41.32 [39.47-42.43]
Minimum MAP per event (mmHg), median [Q1-Q3]	37.13 [34.08-39.63]

Abbreviations: BP, blood pressure; CS, ClearSight continuous BP device; MAP, mean arterial pressure.
Statistic presented as median [first quartile, third quartile] or number of patients (%).
AUT: The AUT is calculated as the ‘depth of hypotension below the threshold defined as a MAP (chosen threshold) mmHg’ x ‘time spent below MAP (chosen threshold) mmHg in minute. TWA: Time Weighted Average Area Under Threshold; TWA= (depth hypotension below MAP threshold in mmHg x time spent below MAP threshold in minutes. Subsequently the formula

of TWA in hypotension is as follows: ‘AUT’ / ‘total duration of measurement period in minutes’. The units for the AUT are mmHg*min and the units for TWA are mmHg.
